# Contribution of Gut Microbiota to Immune Tolerance in Infants

**DOI:** 10.1155/2021/7823316

**Published:** 2021-12-28

**Authors:** Constanza S. Méndez, Susan M. Bueno, Alexis M. Kalergis

**Affiliations:** ^1^Carrera de Nutrición y Dietética, Departamento de Ciencias de la Salud, Facultad de Medicina, Pontificia Universidad Católica de Chile, Chile; ^2^Millennium Institute on Immunology and Immunotherapy, Departamento de Genética Molecular y Microbiología, Facultad de Ciencias Biológicas, Pontificia Universidad Católica de Chile, Santiago, Chile; ^3^Departamento de Endocrinología, Facultad de Medicina, Escuela de Medicina, Pontificia Universidad Católica de Chile, Santiago, Chile

## Abstract

The prevalence of food allergy has increased in recent years, especially among the pediatric population. Differences in the gut microbiota composition between children with FA and healthy children have brought this topic into the spotlight as a possible explanation for the increase in FA. The gut microbiota characteristics are acquired through environmental interactions starting early in life, such as type of delivery during birth and breastfeeding. The microbiota features may be shaped by a plethora of immunomodulatory mechanisms, including a predominant role of Tregs and the transcription factor FOXP3. Additionally, a pivotal role has been given to vitamin A and butyrate, the main anti-inflammatory metabolite. These observations have led to the study and development of therapies oriented to modifying the microbiota and metabolite profiles, such as the use of pre- and probiotics and the determination of their capacity to induce tolerance to allergens that are relevant to FA. To date, evidence supporting these approaches in humans is scarce and inconclusive. Larger cohorts and dose-titration studies are mandatory to evaluate whether the observed changes in gut microbiota composition reflect medical recovery and increased tolerance in pediatric patients with FA. In this article, we discuss the establishment of the microbiota, the immunological mechanisms that regulate the microbiota of children with food allergies, and the evidence in research focused on its regulation as a means to achieve tolerance to food allergens.

## 1. Introduction

Food allergy (FA) or food allergies are pathologies triggered by exposure to food allergens [1]. The prevalence of immunoglobulin (Ig) E-mediated FA in children has increased in recent years, with figures ranging from 1 to 2.53% in the USA [2] and Canada [3] to 5.5% in Chile [4]. Higher proportions are observed in self-report studies, reaching up to 25% in some regions [5]. The most common allergens include peanuts, walnuts, eggs, milk, fish, and soy, varying between countries and age groups [3, 6].

Risk factors for the development of FA include vitamin D deficiency, delayed exposure to food allergens, reduced exposure to microorganisms (as suggested by the hygiene hypothesis), and changes in the microbiota [7]. The gut microbiota corresponds to the group of microorganisms that colonize the intestine [8]. The loss of the gut microbiota homeostasis due to changes in their relative abundance and diversity is known as dysbiosis [9]. This condition has been observed in children with FA, whose gut microbiota profiles differ from those without FA [10]. Food allergies developed by mechanisms not mediated by IgE, present in approximately one-third of the population, also present differences in the intestinal microbiota, and a greater relative abundance of Bacteroides and Alistipes has been observed, in addition to presenting changes associated with probiotic supplementation. Therefore, the alteration of the microbiota would not be associated only with IgE [11].

Currently, the only available treatment for FA consists of the strict exclusion of the allergen from the diet. Nevertheless, this approach may impact the nutritional state of the patient depending on the type and number of allergens involved and the age of the patient at the time of the diagnosis [12]. Therefore, it is necessary to explore novel therapies to induce food-specific immune tolerance and decrease FA symptoms in these patients.

## 2. Differences between the Adult and Infant Immune Systems

The immune system of infants is thought to be under active development and training, making it inherently susceptible to react to microbial agents and generate atopic reactions [13]. Throughout neonatal life, the immune system of the infant relies on the immunity of the mother transferred through the placenta, the exposure during childbirth (the birth canal), and breastfeeding [14]. Although hereditary factors also influence the type of immune response that an infant may develop [15], several studies have suggested that nonhereditary factors are the most relevant for shaping the immune system and developing immunity during the first year of life [16]. For example, a recent study on twins characterized 204 immunity-related parameters and showed that 77% of them were heavily influenced by nonhereditary factors [17]. Additionally, it has been reported that immune cells from infants possess high intraindividual heterogeneity, opposed to what has been described for adults [18]. This observation highlights the relevance of environmental exposure during neonatal life [19].

Most of the components of the immune system of newborns are formed but immature in their function [20]. Regarding cell types, neutrophils appear increased in the fetus but fall to levels that will prevail in adulthood a few days after birth, cytotoxic T lymphocytes present low activity compared to adults, and monocytes and macrophages also appear immature [21]. Valiathan et al. measure the concentration of immune cells in different age groups and observed a predominance of lymphocytes, platelets, and B cells that decrease significantly with age, neutrophils and CD8^+^ T cells increase in adulthood, and natural killer (NK) cells, which are part of the innate immune response, increase mainly in adolescence [22].

Compared to adults, mononuclear cells from children display a reduced capacity to secrete IL-12p70, which plays a pivotal role in the polarization of Th1 cells [23]. Consequently, children show a predominantly Th2 immune response [24].

Functional B and T cells in the gastrointestinal tract are newly expressed at 12 weeks of the newborn, not with the maturity present in adults. However, the transforming growth factor-*β* (TGF-*β*) favors the expression of Tregs [25].

Breast milk plays a fundamental role in the maturation of the immune system since it supplies components that may be absent or immature, such as IgA that is absent, therefore the only source is breast milk, and others such as IgG that has been identified that by being associated with food allergens improves their tolerance, reinforcing that the introduction of allergens together with breast milk would be a protective factor against FA [26].

Due to environmental stimulation, adults (previously exposed to tobacco smoke, altered nutritional status, diabetes, dyslipidemia, or insulin resistance) may display a predominantly proinflammatory immune profile [27].

The differences between the adult and the infant immune system will determine the development of the immune response. Of particular relevance is maternal immunity during gestation and environmental exposure during the neonatal and infant life [28].

## 3. Development of the Microbiota in Infants

**Figure 1:**
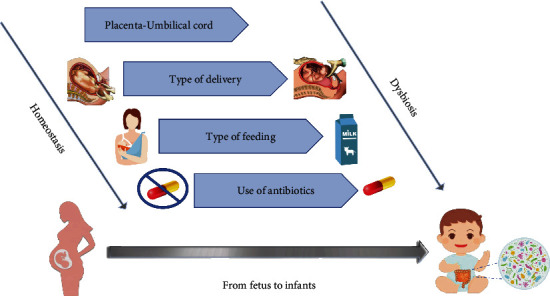
Factors that determine the development of the microbiota in infants. The development of the microbiota begins during pregnancy and continues after birth with exposure to environmental factors. To the left of the figure are factors associated with homeostasis. To the right of the figure are factors related to dysbiosis.

Early gut microbiota marks the health of the individual in later life, where it has been associated with pathological conditions triggered later during old age [29] (Figure 1). However, little is known on the specific time point at which disease-generating dysbiosis is generated, the exact composition of a “healthy” microbiota, and whether its alteration is due to pathological conditions, such as food allergies [30], irritable bowel syndrome [31], celiac disease [32], or physiological status, including pregnancy, dietary changes, or age (Figure 1) [33, 34].

### 3.1. Factors Associated with the Prenatal and Neonatal Periods

The microbiota colonization begins during gestation, a period in which maternal nutrition and health status influence the type of microorganisms present in the placenta and umbilical cord [35–37]. These factors contribute to the generation of a microbiota profile even before childbirth [38]. Although it was previously thought that the gestational period hosted a sterile environment [39], it is currently known that the establishment of the gut microbiota in infants initiates during pregnancy by the presence of maternal microorganisms that translocate through the vagina, maternal gut, placental tissue, and meconium, thus discarding the concept of a sterile placenta [40, 41].

A report studied the composition of the microbiota of the placenta, amniotic fluid, meconium, newborn stool samples, and maternal stool samples from patients that underwent optional C-sections, but there was no similarity between the microbial populations of amniotic fluid and placenta, where there were nondiverse populations of *Proteobacteria* (*Enterobacter* and *Escherichia/Shigella*), also present in colostrum and meconium but less abundantly [42]. Contrary to this report, another study reported no microbiota in the placenta or amniotic fluid in C-sections [41]. However, 100% of the patients received prophylactic antibiotic treatment that may have affected the results of this study [41, 43]. It remains a matter of discussion whether the presence of placental or amniotic microorganisms results from the development of the fetus or is only detected following microbial translocation from the mother to the fetus [42].

The mode of delivery is known to impact the development of the gut microbiota [44]. A sevenfold higher abundance in *Bifidobacterium*, *Proteobacteria*, and the genus *Enterobacter-Escherichia-Klebsiella*, *Clostridium*, and *Enterococcus* has been reported in newborns delivered by vaginal birth compared to C-section, the latter being deprived of exposure to the birth canal, thus presenting a greater abundance of *Bacteroidetes* and a reduced fraction of *Streptococcus* [45]. A second study from Korea compared the microbiota of stool samples from infants born of vaginal birth and C-section at 3, 7, and 14 days postpartum. Reduced bacterial diversity was observed with time. Microbiota composition of C-section infants varied from day 14, suggesting that this mode of delivery is associated with the delayed establishment of the gut microbiota, associated with obesity and asthma later in life [46]. Newborns via vaginal birth displayed a higher abundance of *Bifidobacterium*, *Bacteroides*, *Lactobacillus*, and *Lachnospiraceae* at day 7 compared to C-section, in which *Enterococcaceae* and *Enterobacteriaceae* were more abundant [46].

### 3.2. Factors Associated with the Postnatal Period

Breastfeeding also influences microbiota development because breast milk contains bioactive compounds, such as oligosaccharides that nourish intestinal bacteria, molding the gut microbiota [47]. A recent study of Chilean newborns compared the intestinal microbiota of infants fed with breast milk to that of infants fed with formula at months 1 and 3 and showed that the microbiota of infants fed with breast milk displayed a higher *Bacteroidetes* abundance [48]. In contrast, those fed with formula showed a higher proportion of *Firmicutes*. At the genus level, the *Enterococcus*, *Streptococcus*, *Enterobacter*, *Lactococcus*, and *Propionibacterium* communities were enriched in the breast milk group at the first month. These differences in bacterial diversity were no longer present in the third month [48, 49]. Another study evaluated the fecal microbiota composition at 40 days, 3 months, and 6 months postpartum; it reported that *Bifidobacterium* and *Enterobacteriaceae* were the most abundant in breastfed infants at all time points and were more abundant in this group than formula-fed infants; to differ the formula group, *Streptococcus* and *Enterococcus* were the most abundant [50]. In the same study, when evaluating the introduction of complementary feeding, the abundance of *Bacteroides* increased in the formula group, which did not occur in breastfed infants, which was associated with increased diversity of the microbiota in the formula group [50].

## 4. Mechanisms of Microbiota Dysbiosis Associated with Food Allergy

The association between microbiota and FA was first reported in germ-free mice displaying elevated IgE levels in the intestinal mucosa without additional alterations of the other immunoglobulins [51]. Based on this observation, it is believed that the microbiota may contribute to maintaining the homeostasis of IgE and the control of allergic responses triggered by these immunoglobulins [52]. Further, the population of microorganisms in the intestine of mice with FA would favor a Th2-type response, shifting the balance from Th1 to Th2 immune profile [53]. It was shown that *Citrobacter* sp., abundant in FA models, induces the expression of IL-33, promoting a Th2-type immune response [54].

Short-chain fatty acids (SCFA) are fundamental to the microbiota, as they provide metabolites that serve as nourishment to bacterial communities. Of great importance are butyrate, propionate, and acetate that result from the fermentation of dietary carbohydrates by intestinal bacteria [55, 56]. In infants younger than 6 months, dietary carbohydrates correspond to breast milk oligosaccharides, which are not physiologically digested by the infant but serve as sustenance for the microbiota, mainly the genus *Bifidobacterium* and *Bacteroides* [57].

The most prevalent SCFA in 3-month-old infants is acetate, where it can be found between 70 and 90% of the total SCFA, followed by propionate and butyrate, with an increase of up to 4 times with the start of feeding, complementary at 6 months [58, 59]. Although it is not the metabolite present in the greatest quantity, butyrate has been more studied and associated with the production of regulating microorganisms of the microbiota and with a lower probability of developing asthma and food allergies in infants [60, 61].

Nilsen et al. observed correlations between bacterial species and relative amount of SCFA, for example, the presence of the *E. rectocele* and *F. prausnitzii* network with a higher relative abundance of butyrate measured at 12 months of the infant. These networks have been identified as prominent producers of butyrate in adults. The authors suggest that eating habits between 6 and 12 months are crucial for establishing the adult microbiota [58]. Butyrate fulfills regulatory functions in the immune system as an inhibitor of histone deacetylase, reducing the release of proinflammatory cytosines through G protein-coupled receptors, among other pathways. In addition, this molecule also contributes to regulating the function of T cells. Therefore, factors such as incorporated foods and lifestyles during the early stages could affect immune mechanisms in later stages [60].

### 4.1. Effect of the Microbiota on the Innate Immune System

Although the epithelial barrier prevents the contact of allergens with the immune system, in some cases, the antigens can cross this barrier and cause sensitivity to food allergens [62]. During the innate immune response, at the skin level, exposure to the allergen in a defective epithelial barrier causes keratinocytes to synthesize alarmins such as IL-33, thymic stromal lymphopoietin (TSLP), and IL-25, cytokines with the function of sending an “alarm signal” and activating type 2 innate lymphoid cells (ILC-2) [63–65]. ILC-2 will stimulate the production of Th2 cytokines, especially IL-5, IL-14, IL-4, and IL-9, which are characteristic of food allergies [66, 67]. A study carried out in mouse models for FA showed that mice deficient in the IL-33 receptor (IL-33R) do not develop FA because they cannot generate ILC-2 differentiation, and IL-4 is capable of suppressing Treg differentiation through increased mast cell activation [68]. Therefore, differentiation to ILC2 contributes to the development of FA by promoting the bias of the immune response towards a Th2 and proinflammatory phenotype.

The intestinal microbiota is capable of inducing the innate immune response [69, 70]. A recent study evaluating the effect of SCFA on the innate lymphoid cell (ILC) response in mice found that SCFA administration suppressed the IL-33-induced ILC2 response in WT mice and Ffar2-/- mice [71].

### 4.2. Butyrate as the Main Metabolite in Microbiota Regulation

**Figure 2:**
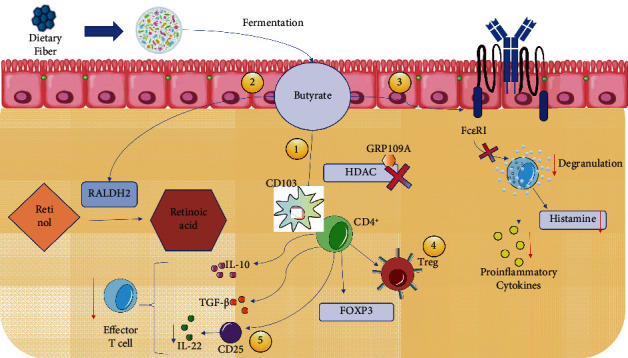
Intestinal microbiota immunological mechanism against FA. 1: inhibits HDAC through the GRP109A receptor, acting on CD103 and promoting Treg differentiation; 2: induces RALPH activity, also acting on CD103; 3: inhibits the Fc*ε*RI receptor, decreasing degranulation and thereby the expression of proinflammatory cytokines; 4: Treg expresses FOXP3 and synthesizes inhibitory cytokines such as IL-10 and TGF-*β*; 5: CD25, expressed by Treg FOXP3, is capable of decreasing effector T cell proliferation. All these mechanisms contribute to microbiota homeostasis and the gaining of tolerance.

Butyrate has been identified as the metabolite with the most significant effect on immunity due to the capacity of this molecule to promote anti-inflammatory pathways by inducing CD4^+^ T cell differentiation into the Treg cells (with a fundamental role in allergen tolerance) mediated by the inhibition of histone deacetylases (HDAC) through the GPR109A receptor expressed on the surface of these cells, which among all metabolites is activated only by butyrate and also by niacin (Figure 2) [66]. This receptor acts on CD103^+^ cells capable of promoting the proliferation of Treg cells [68, 69].

In addition, butyrate can induce the activity of retina dehydrogenase (RALDH2), an enzyme responsible for the conversion of vitamin A into retinoic acid (RA) and that is expressed by CD103^+^ DCs. It has been suggested that RALDH2 would promote Treg cell differentiation, which favors oral tolerance (Figure 2) [70].

It has been observed that during intestinal pathologies, the presence of a proinflammatory cytokine in conjunction with retinoic acid leads to the loss of tolerance, such as IL-15; there is a lower abundance of butyrate-producing bacteria and therefore a lower concentration of the total levels of the metabolite in the epithelium, promoting microbiota dysbiosis and decreasing Treg differentiation [67, 71]. Finally, butyrate could inhibit the high-affinity IgE receptor- (Fc*ε*RI-) triggered degranulation of mast cells, which leads to a decrease in the release of inflammatory mediators and histamine, reducing the development of allergic reactions [72].

### 4.3. Effect of Treg Cells on Microbiota

Treg cells have an anti-inflammatory effect that contributes to immune tolerance, counteracting the function of follicular T helper (THF) cells, which are needed for IgE synthesis. Therefore, Treg cells can downmodulate IgE synthesis and reduce allergic reactions [73].

In addition, it has been observed in mouse models that the synthesis of colonic Treg cells increases in mice colonized with benign microbiota compared to germ-free mice, which is why they would be highly related to the gut microbiota [74]. Following this observation, other researchers identified a lower frequency of Treg cells in mice treated with vancomycin, an antibiotic targeting Gram-positive bacteria, compared to mice that were administered polymyxin, associated with Gram-negative bacteria, suggesting a predominant role of Gram (+) in the accumulation of Treg, such as *Clostridium* [75].

A factor that has been identified as an important inducer of allergen tolerance is forkhead box P3 (FOXP3), a transcription factor expressed by FOXP3+Treg cells together with CD25 (IL-2 high-affinity receptor), and according to research, a greater inflammatory response associated with allergies has been observed in knockout FOXP3 mice [76, 77]. Furthermore, significant inflammatory responses have already been observed in FOXP3 KO mice [78], while children with IgE-mediated FA have also shown a lower expression of FOXP3 as compared to healthy controls [79].

Tregs can also modulate the immune response by means of the production of IL-10 and TGF-*β*, which are cytokines that in general suppress immune responses [76]. In addition, a mechanism associated with IL-2 has been identified, through the supplementation of IL-2 to mice with peanut allergy, where the supplemented mice presented increased function and number of Treg cells, therefore prevention of food allergy for a period of 7 months [80].

### 4.4. Modulation of the Mastocyte Function by the Microbiota

Th2 lymphocytes activate mast cells after exposure to an allergen, which in turn are responsible for releasing histamine and other mediators of inflammation, such as cytokine tumor necrosis factor alpha (TNF-*α*), and for generating the characteristic symptoms of IgE-mediated FA [81].

The intestinal microbiota also appears to regulate mast cell expression and functionality, as observed in a study with mice free of minor germs and low mast cell functionality; however, the mechanism is not yet clear [82]. Another proposed mechanism is that acetate produced by *Bifidobacterium spp* induces mast cell apoptosis in mouse mast cells, reducing allergic symptoms [83].

Finally, evaluating therapeutic options, an investigation administered *Bifidobacterium longum* KACC91563 to Balb/c mice with induction of FA and observed an increase in mast cell apoptosis with consequent reduction of allergy symptoms, providing additional evidence for the role of these cells in the regulation of FA [84].

## 5. Future Treatments for FA with a Focus on Microbiota

Currently, available evidence addressing the impact of the microbiota on FA regulation encouraged researchers to formulate interventions with compounds that can correct the dysbiosis state, favoring the improvement of symptoms and resulting in tolerance to allergens [85].

Probiotics and prebiotics with different fiber types are some of the current approaches to improve microbiota composition and reduce dysbiosis (Table 1).

The focus on the microbiota has been present for several years, starting with interventions carried out using yogurt to reduce gastrointestinal symptoms, which, however, had low therapeutic action [86, 87]. Nonetheless, latter experiences were performed with the first uses of probiotic strains, while today, other microorganisms that can regulate the intestinal environment have been evaluated [88].

Research performed on animals has successfully used various probiotics and prebiotics at different times during childhood, yielding promising results in modifying the microbiota towards a healthy profile and decreasing IgE levels, proinflammatory cytokines, and anaphylaxis symptoms [89–92]. However, in humans, the type and dose of probiotic or prebiotic and the proper time for their prescription have yet to be defined.

### 5.1. Prebiotics

Prebiotics are defined as a substrate for host microorganisms to which health benefits are attributed [93]. In animal models, a study performed in fiber-supplemented mice showed a reduction in anaphylaxis symptoms and greater tolerance to allergens with the intervention [70].

In humans, prebiotics used to modulate the microbiota are short-chain galactooligosaccharides (GOS) and short-chain fructooligosaccharides (FOS), and their use as supplements in milk formula has been evaluated for food allergy prevention in susceptible infants [94] and in pregnant women to prevent infant allergy [95].

Milk formula supplementation with prebiotics has been evaluated from birth in children with high risk of atopy, where the appearance of symptoms such as atopic dermatitis and infectious episodes were evaluated in a prospective cohort, where infants were supplemented fewer infectious episodes and cumulative incidence of atopic dermatitis [96].

In addition, in infants with a low risk of atopy in follow-up up to 12 months, supplementation with GOS/FOS resulted in a lower incidence of atopic dermatitis compared to infants without prebiotics and a tendency for the clinical presentation to be less severe [97].

Using other prebiotics, children with atopic dermatitis were supplemented using Kestosa, a FOS capable of stimulating the activity of Bifidobacteria, and observed a decrease in clinical symptoms [98], and a clinical trial used a mixture of polydextrose (a prebiotic present in breast milk) and GOS in infants at high risk of atopy; however, no differences were observed in the development of atopic dermatitis between the groups at 2 years of age [99]. Although reviews have been published on the effect of prebiotics on the development of immunological diseases in infants, so far, no interventions have been carried out where the use of prebiotics is considered a scientifically proven treatment for food allergy [94, 100].

### 5.2. Probiotics

Probiotics are defined as living microorganisms that, in suitable doses, provide benefits for their host [101]. Unlike prebiotics, the use of probiotics in research has been more extensive, and the most commonly studied strain is Lactobacillus GG (LGG), a component of dairy formulas for the pediatric population. Although research has evaluated changes in the gut microbiota profile in infants after probiotic use, not all have followed up to assess changes in clinical symptoms [102].

A prospective clinical trial in 329 children with cow's milk allergy (CMA) evaluated the acquisition of tolerance in children with ingestion of a formula extensively hydrolyzed with LGG probiotics, and 80% of the children treated with this formula had acquired tolerance at 12 months, with significant differences at 12 months as compared to the other types of formula without probiotics [103].

However, opposite results have been observed for some studies, in which no acquisition of early immune tolerance has been observed [104]. A systematic review that sought to evaluate the efficacy of probiotic supplementation in CMA in children under 5 years of age observed a higher proportion of children who reached tolerance after 36 months with a RR 1.47, with no significant difference at 6 and 12 months [105].

In addition, the effect on tolerance of oral immunotherapy (ITO) in coadministration with probiotics has been studied in children with peanut allergy, where a sustained lack of response was observed in 82% of children operated after 2-5 weeks; however, this study does not evaluate whether the effect is maintained when probiotics are withdrawn [106]. For that purpose, a protocol for a randomized controlled trial in phase 2 was recently published to intervene with OTI and probiotics in children with peanut allergy and to be able to evaluate the contribution that probiotics have in this therapy [107].

Other studies have shown similar results in modifying intestinal microbiota profiles; however, they differ in the probiotics used and the dosage, and some are performed in a small number of patients (Table 1). Due to these limitations, it is not yet possible to make clinical recommendations regarding the use and effectiveness of probiotics as a treatment for food allergy.

## 6. Concluding Remarks

Human research on the use of prebiotics or probiotics to modify the intestinal microbiota and therefore induce FA tolerance is limited for probiotics or absent in the case of prebiotics and with a low number of participants. The latter does not allow making clinically applicable recommendations. Although there is a trend leaning towards research on LGG 74-77, other populations should be identified that may significantly impact the microbiota and have a more clinically evident benefit.

The lack of research on exclusively breastfed children under 6 months is striking, while the main recommendation is of this being a protective factor and an immune mediator [108], in addition to providing FOS/GOS as a part of its nutritional composition [109]. Although the studies carried out have not evidenced adverse effects or tolerance to supplementation, more robust evidence is needed to support the use of prebiotics and probiotics as a general recommendation.

## 7. Conclusion

The impact of the intestinal microbiota on the development of FA is well known. However, the mechanisms involved are not yet clear. The modification of intestinal microbiota has shown promise in animal models regarding both immunological and clinical parameters. However, human research is still scarce and should be carried out with a more significant number of participants. Probiotics and prebiotics are proposed as innovative, safe, and economical therapy once the most effective agents and their appropriate doses are precisely identified.

## Figures and Tables

**Table 1:** Evidence regarding microbiota interventions in children with FA.

Type of FA	Age	*n*	Prebiotic/probiotic	Form of administration	Doses	Main result	Reference
CMA	10-25 months	12	*Lactobacillus rhamnosus GG*	Extensively hydrolyzed commercial milk formula supplement	N/A	Enriched fecal microbiota with LGG formula consumption	[110]
CMA	4-6 years	330	*Lactobacillus rhamnosus GG*	Extensively hydrolyzed commercial formula	LGG 2.5 × 10^7^ to 5 × 10^8^ CFU/g	Less functional dyspepsia, functional constipation, and functional abdominal pain in children with probiotics	[111]
CMA	1-12 months	220	*Lactobacillus rhamnosus GG*	Extensively hydrolyzed commercial formula	N/A	Less allergy-associated symptoms and faster tolerance acquisition at 12, 24, and 36 months	[112]
CMA	1-12 months	39	*Lactobacillus rhamnosus GG*	Extensively hydrolyzed commercial formula	4.5 × 10^7^‐8.5 × 10^7^ CFU by gram of powder	Higher production of butyrate and related to higher production of butyrate in tolerant patients	[113]
Peanut allergy	1-10 years	62 children	Coadministration of *Lactobacillus rhamnosus* CGMCC 1.3724 (NCC4007) and oral immunotherapy with peanuts	Lyophilized powder	2 × 10^10^ CFU once a day together with peanut OIT for 18 months	Reduced peanut sensitization in combination with immunotherapy	[106]
CMA	<6 months	119	*Lactobacillus casei* CRL431 and *Bifidobacterium lactis* Bb-12	Extensively hydrolyzed commercial formula	N/A	No differences were observed regarding severity of atopic dermatitis	[114]
CMA	<1 year	260	*Lactobacillus rhamnosus GG*	Extensively hydrolyzed commercial formula	N/A	Earlier acquisition of tolerance in supplemented children	[103]
CMA	3-12 months	60	*Bifidobacterium lactis* BB-12 and *Streptococcus thermophilus* TH-4	Extensively hydrolyzed commercial formula	*Bifidobacterium lactis* BB-12 (1 × 10^9^ CFU) and *Streptococcus thermophilus* TH-4 (1 × 10^8^ CFU)	Reduced clinical symptoms with supplementation	[115]
CMA	0-12 months	26	*Lactobacillus rhamnosus GG*	Extensively hydrolyzed commercial formula	2.50 × 10^7^ to 5 × 10^8^ CFU/g	Higher decrease of calprotectin and reduction of fecal hematochezia in supplemented participants	[116]
CMA	<6 months	111	*Lactobacillus casei CRL431 (Lactobacillus paracasei subspecies paracasei) and Bifidobacterium lactis Bb-12*	Extensively hydrolyzed formula	1 × 10^7^ colony-forming units/g formula for each of the probiotic bacteria used	No difference was observed in the age of acquisition of allergen tolerance	[104]

## Abbreviations

CFU: Colony-forming unit
CMA: Cow's milk allergy
FA: Food allergy
Fc*ε*RI: High-affinity IgE receptor
FOS: Short-chain fructooligosaccharides
FOXP3: Forkhead box P3
GOS: Galactooligosaccharides
HDAC: Histone deacetylases
IG: Immunoglobulin
IL-33R: IL-33 receptor
ILC: Innate lymphoid cells
ILC-2: Type 2 innate lymphoid cells
ITO: Oral immunotherapy
LGG: Lactobacillus GG
NK: Natural killer
RA: Retinoic acid
RALDH2: Retina dehydrogenase
SCFA: Short-chain fatty acids
TGF-*β*: Transforming growth factor-*β*
THF: Follicular T helper cells
TNF-*α*: Tumor necrosis factor alpha
Tregs: Regulatory T cells
TSLP: Thymic stromal lymphopoietin.
